# Natural History Specimens

**Published:** 1860-06

**Authors:** 


*Natural History Specimens.*—A pamphlet has been issued, giving instructions to persons who may be willing to take the trouble to send specimens of natural history, such as minerals, skins of animals, of birds, snakes, etc., to the great national collection of these specimens which is being made by the Smithsonian Institution. It is requested that the most common species of each neighborhood should be forwarded. The pamphlet of instructions will doubtless be sent to any one who may write for it to Professor Joseph Henry, the Secretary.—*Scientific American.*

